# Prize Essay on Nostrums

**Published:** 1850-11

**Authors:** 


*Prize Essay on Nostrums.*—The commitee of the New York State Medical Society, on prize dissertations, reported at its last session the reception of one essay on the subject for which the prize was offered last year. This being not in accordance with the spirit and requirements of the resolution under which it was offered, they recommended the passage of the following resolution, which was adopted:—

*Resolved*, That a prize of twenty dollars be offered for the best essay "on the pernicious influences of nostrums and secret remedies on the health and morals of the community." Said essay to consist of not less than sixteen pages, or more than twenty of the Transactions, to be adapted for popular, rather than professional instruction. The essays to be transmitted to the Secretary of the Society, before the 1st of January, 1851.—*N. Y. Med. Journal.*

